# Gamified Motor Training With Tangible Robots in Older Adults: A Feasibility Study and Comparison With the Young

**DOI:** 10.3389/fnagi.2020.00059

**Published:** 2020-04-03

**Authors:** Arzu Guneysu Ozgur, Maximilian J. Wessel, Jennifer K. Olsen, Wafa Johal, Ayberk Ozgur, Friedhelm C. Hummel, Pierre Dillenbourg

**Affiliations:** ^1^Computer Human Interaction in Learning and Instruction Lab, Swiss Federal Institute of Technology (EPFL), Lausanne, Switzerland; ^2^Defitech Chair of Clinical Neuroengineering, Center for Neuroprosthetics (CNP) and Brain Mind Institute (BMI), Swiss Federal Institute of Technology (EPFL), Geneva, Switzerland; ^3^Defitech Chair of Clinical Neuroengineering, Clinique Romande de Réadaptation, Center for Neuroprosthetics (CNP) and Brain Mind Institute (BMI), Swiss Federal Institute of Technology (EPFL Valais), Sion, Switzerland; ^4^BIOROB Lab, EPFL, Lausanne, Switzerland; ^5^Clinical Neuroscience, University of Geneva Medical School, Geneva, Switzerland

**Keywords:** tangible robots, healthy aging, gamified exercise, robotic exercise, motor learning, transfer learning, task performance and analysis

## Abstract

**Background:** An increasing lifespan and the resulting change in our expectations of later life stages are dependent on a good health state. This emphasizes the importance of the development of strategies to further strengthen healthy aging. One important aspect of good health in later life stages is sustained skilled motor function.

**Objective:** Here, we tested the effectiveness of robotic upper limb motor training in a game-like scenario assessing game-based learning and its transfer potential.

**Methods:** Thirty-six healthy participants (*n* = 18 elderly participants, *n* = 18 young controls) trained with a Pacman-like game using a hand-held Cellulo robot on 2 consecutive days. The game-related movements were conducted on a printed map displaying a maze and targets that had to be collected. Gradually, the task difficulty was adjusted between games by modifying or adding different game elements (e.g., speed and number of chasing ghosts, additional rules, and haptic feedback). Transfer was assessed by scoring simple robot manipulation on two different trajectories.

**Results:** Elderly participants were able to improve their game performance over time [*t*_(874)_ = 2.97, *p* < 0.01]. The applied game elements had similar effects on both age groups. Importantly, the game-based learning was transferable to simple robot manipulation that resembles activities of daily life. Only minor age-related differences were present (smaller overall learning gain and different effects of the wall-crash penalty rule in the elderly group).

**Conclusions:** Gamified motor training with the Cellulo system has the potential to translate into an efficient and relatively low-cost robotic motor training tool for promoting upper limb function to promote healthy aging.

## 1. Introduction

Life expectancy is constantly rising. According to the current World Health Organization's statistics, the global life expectancy at birth has increased from 64 years in 1990 to 71 years in 2013 (World Health Organization, 2016). For many individuals, this opens up for new opportunities, such as starting a new career, continuing education, or pursuing a neglected passion at later stages in life (Beard et al., 2016). To be able to benefit from these opportunities, an important prerequisite is healthy aging, which corresponds to good physical and especially mental health. One important aspect of healthy aging is the capacity to acquire and maintain skilled motor abilities at a later life stage to be able to adjust to the challenges and requirements of the changes of daily life, such as the constant adjustment to novel communication devices. These skills enable us to interact with our environment and allow functional independence for many activities of daily living. One key factor for functional independence in the elderly are skilled upper limb functions and the acquisition of novel motor skills (Scherder et al., 2008). This feature typically exhibits age-related performance declines, which can be characterized by slowing of movement (Ketcham et al., 2002), increased variability (Cooke et al., 1989), and coordination difficulties, especially at increased complexity, e.g., for multi-joint movements (Seidler et al., 2002); to read a review please see Seidler et al. (2010). Moreover, elderly people often change their strategy to achieve their goals, favoring movement accuracy over speed (Salthouse, 1979).

Motor training protocols can partially ameliorate the described deficits common in the elderly population (Levin et al., 2017) via the recruitment of motor learning mechanisms and promotion of neuroplasticity (Cai et al., 2014). A current constraint is that motor learning abilities are often reduced in the elderly population; for a review please see King et al. (2013). This restricted ability can be evident by reduced learning rates and magnitude, especially in early learning stages (fast learning) (Curran, 1997; Shea et al., 2006; Zimerman et al., 2013), impaired in-between session learning (offline learning) (Spencer et al., 2007), or slower relearning after a longer interval without training (savings) (Rodrigue et al., 2005).

In this paper, we evaluated the combination of a motor training method using tangible robots in addition to the application of gamification strategies. For the robotized motor training, small-sized, graspable, haptic-enabled Cellulo robots were used (Özgür et al., 2017b), and these operate on printed paper sheets (Guneysu Ozgur et al., 2018). These tangible robots serve as a “computer-mouse-like” interface to interact with printed elements on paper, directing trajectories, and can provide visual and haptic feedback. In the present motor training approach, by using tangible robots both as game agents and objects to be moved, we aimed to provide an intuitive easy-to-use and easy-to-set-up system for motor training. Recent studies showed that utilizing tangible objects that the elderly are familiar with and providing a tangible interface with simplified elements may facilitate learning of using these technologies among elderly (Apted et al., 2006; Hung et al., 2016; Garcia-Sanjuan et al., 2017; Wang et al., 2017; Guneysu Ozgur et al., 2018). Furthermore, making the learning of using these devices easier might be crucial, since the lack of familiarity and the steep learning curve of using training devices may reduce the training efficiency among elderly users (Aarhus et al., 2011; Gerling et al., 2011; Guneysu Ozgur et al., 2018). Direct contact via touch interfaces is proposed to provide lower cognitive loads and a more suitable and intuitive alternative, especially for aging users (Garcia-Sanjuan et al., 2017; Guneysu Ozgur et al., 2018).

The Cellulo system also provides the opportunity to integrate gamification aspects within the motor training sessions. These gamification aspects have been conceptualized as the use of design elements characteristic for games in non-game contexts (Deterding et al., 2013). By this means, the participants are exposed to typical elements of game playing, such as rule-based and goal-oriented behavior, problem-solving, feedback, or competition (Sailer et al., 2017). Furthermore, gamification can increase participants engagement (Looyestyn et al., 2017).

We hypothesized that, with this combined approach (robotized motor training combined with gamification strategies), age-related constraints in motor skill learning can be partially ameliorated via the enhancement of participants engagement and concurrent training of cognitive processing. For instance, this cognitive training could involve executive (planning, decision-making, and flexibility), attentional (processing speed and divided attention) or memory (implicit learning) domains (Sachdev et al., 2014). By this means, the Cellulo platform may also translate into a rehabilitation tool for neurological patients with an elderly age profile including, for example, stroke.

To pursue our aim, we first assessed the feasibility of a Cellulo-based gameplay with a group of healthy elderly participants, characterized the learning process and compared it to young adults, identified game elements with a different impact in the elderly group, and investigated transfer to simple input device manipulation. In detail, we examined the impact of age group on overall learning, daily learning, overnight learning, and transfer learning, and we also examined the effect of game elements and configurations on the performance. These different analyses were done to answer various research questions:

- RQ1–Overall Learning: Do participants have a better game performance on the second day compared to the first day, and is this performance impacted by age group?
- RQ2–Online Learning: Do participants improve their game performance within a day, and is this performance impacted by age group?
- RQ3–Overnight Learning: Is there evidence of overnight learning operationalized as the participants performing better on the first game of the second day compared to the last game of the first day after having slept in between, and is this performance impacted by age group?
- RQ4–Transfer Learning: RQ4A: Did the game intervention impact participant performance on transfer activities between pre and post-intervention, and is this performance impacted by age group? RQ4B: Is there evidence of overnight learning on transfer activities operationalized as the participants performing better on the first activity of the second day compared to the last activity of the first day after having slept in between, and is this performance impacted by age group?
- RQ5–Impact of the Game Elements: RQ5A: How does each game element impact the performances of different age groups? RQ5B: How do the configurations of game elements impact the performances of different age groups?

Each subsection under the results in section 3 shows the corresponding test results of each research question. Similarly, each corresponding subsection under section 4 includes our discussion for the corresponding results and the state of the art.

## 2. Materials and Methods

### 2.1. Subjects

Thirty-six healthy individuals were included in this study. Among the 18 elderly and 18 young participants, one elderly participant dropped out of the experiment and did not complete the whole gameplays due to time limitations, and two young participants' data were excluded because of missing data. There were several inclusion criteria: (1) right-handedness, (2) normal values of Mini-Mental-State-Examination (>26/30) (Folstein et al., 1975), (3) 18–35 years for the young group, and (4) >60 years for the elderly group. Exclusion criteria were (1) neuropsychiatric diseases, (2) history of seizures, (3) musculoskeletal dysfunction that compromise finger movement, (4) pregnancy, (5) professional musicians or intense professional usage of a computer keyboard, (6) intake of narcotic drugs, and (7) request of not being informed in case of incidental findings. For 2 days of consecutive study and to compensate for travel expenses, each elderly participant was compensated with a gift card worth 50 Swiss Francs for a shopping center, while young participants were paid the same amount with cash. The study protocol was approved by the Cantonal Ethics Committee Vaud, Switzerland (project number 2017-00765). All subjects gave written informed consent in accordance with the Declaration of Helsinki. For further participant characteristics, information of age, gender ratio, and Mini-Mental State Examination (MMSE) assessment scores see Table 1.

**Table 1:** Participants' characteristics.

**Age group**	**Number of subjects**	**Mean age ± SD**	**Mean MMSE ± SD**	**Gender ratio**
Old	17	66.65 ± 5.79	29.18 ± 0.81	4 female/13 male
Young	16	23.38 ± 4.76	29.31 ± 0.79	4 female/12 male

### 2.2. Cellulo Robotic Platform

Cellulo is a small-sized tangible robot that operates on printed paper sheets. Cellulo is accurately 2D-localized in (*x, y*, θ) with sub-millimeter precision, and its magnet-ball drive locomotion system allows it to move and to be moved freely by the user. Its design enables it to be used as a haptic interface to render, for instance, 2D planar force information (Özgür et al., 2017a).

Cellulo robots offer a unique perspective in game design, where mobile, physical game elements can be programmed to act as agents (rival, ally, or neutral), and input devices invoke physical exercise while transparently capturing kinematic data from the user.

The robots are designed to be simple to operate; all robots are connected wirelessly to a mobile device (a tablet or smartphone) that runs the activity and game logic. The current system includes self-localization on the activity sheet covered with a dot pattern (Hostettler et al., 2016), holonomic motion robust against human manipulation (Özgür et al., 2016), six capacitive touch buttons (independently back-illuminated in full RGB that can provide visual feedback), and wireless Bluetooth communication (Özgür et al., 2017b). The platform provides fast (>90 Hz) and accurate localization (sub-mm) of many robots, which can be logged to record all the interactions during the game, such as user motion.

**Figure 1:**
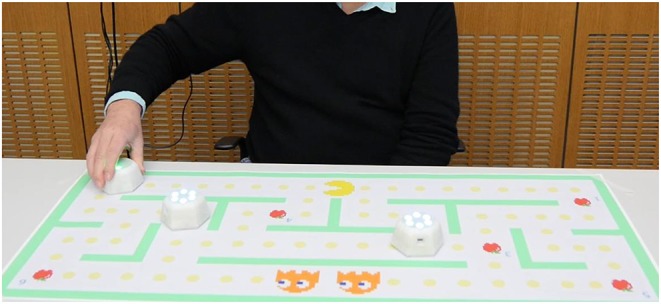
Cellulo robotic platform: elderly participant playing the Pacman game by controlling a Cellulo robot with his right hand. The aim of the game is to collect the fruits by not crashing the walls of the maze (green bars) and by not getting caught by the chasing robots.

The scenery of the activity is printed on paper sheets that can feature any desired graphical game elements defined as active zones. These zones can be associated with specific robot behaviors to design game logic. For instance, in Figure 1, green walls activate assistive haptic behavior of the robot, while fruits represent target objects to be collected in a game. The raw robot positions are also used, for example, for onboard closed-loop motion control (including haptic feedback) and on the external controller for multi-robot formation control. Therefore, an activity is the combination of the paper elements, the robots with particular interaction modalities, and the tablet that runs the activity-specific software. As such, the role of the robots and paper depends on the design of each particular activity.

### 2.3. Pacman Game

In order to test the potential of Cellulo for upper arm motor training regimes, we used an iterative approach to design a game using the robots as game agents and objects. Our first game, inspired by Pacman, was designed iteratively with the participation of stroke, brachial plexus, and cerebral palsy patients (18 in total) and seven therapists in four different therapy centers (Guneysu Ozgur et al., 2018).

In the game, the printed map displays the maze and the fruits to be collected by the player. The player holds the Pacman robot in his/her hand and is chased by one or two autonomous robots that are referred as ghosts. The player is expected to collect all six apples as quickly and precisely as possible to finish the game (we define precision as not crashing into the maze walls). The active agents (called ghosts) chase the user's robot during the game in order to catch it; all previously collected apples are lost and the ghosts return to their initial positions if this happens. The game finishes when all six apples are collected.

**Figure 2:**
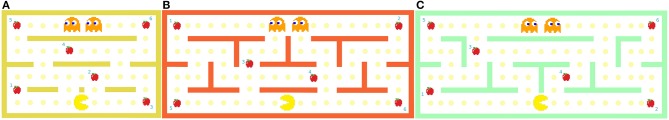
Three maps with different mazes and sizes. The game was played on three different maps with an ABBC order, and the maps are composed of different combinations of **(A)** a yellow map, **(B)** an orange map, and **(C)** a green map. The first map was played on the first half of day 1, the second map on the second half of day 1 and the first half of day 2, and third map on the second half of day 2.

Several game elements are designed for tuning speed, accuracy, range of motion, and challenge of the game play. These game elements are:

- Different maps with different maze designs or sizes (orange and green maps with size of 96 × 42 cm and yellow map with size of 62 × 42 cm, see Figure 2 for these three different maps with different maze designs)
- One or two ghost robot(s) chasing the Pacman
- Speed of the chasing robot(s) (mm/s)
- Turn rule: the user can only collect the fruits by rotating the robot on top of them, it can be switched on/off
- Cross border penalty rule: the user loses the last eaten fruit when he/she crashes into a wall, it can be switched on/off
- Haptic feedback: the Pacman robot provides haptic informative assistance when the user crashes into a wall, it can be switched on/off.

These parameters, as described in Guneysu Ozgur et al. (2018), allow us to adapt the difficulty of the game according to the ability of the player.

### 2.4. Pre-post Tests

Like other forms of real-world learning, one of the important desired learning goals of a motor training is transfer to other contexts and tasks (Schmidt and Bjork, 1992; Krakauer, 2006; Gudberg and Johansen-Berg, 2015). In order to investigate the transfer of the learning to a simple device manipulation, we designed two different line following activities as pre-post tests.

Both activities are simple robot manipulation activities on a defined trajectory, activities similar to holding a towel and cleaning a table-top surface as a daily activity. The straight pre-post test activity includes a trajectory with a start and an end point including sharp rectangular turns similar to the Cellulo Pacman Game maze. On the other hand, the curvy pre-post test activity includes a curved trajectory including smoother direction changes with a start and an end point that is closer to daily life motions, such as wiping a table. The approximate distance that is traveled on the curvy trajectory is 310 cm in length while it is 390 cm on the straight one. However, the range of motion limits of both maps are the same: ~90 cm on the horizontal axis and ~38 cm on the vertical axis of the maps. Both test maps can be seen in **Figure 4**.

During the activity, one robot automatically goes to the start point, and the user holds the robot to manipulate it on the path through the trajectory from the start point to the finish trophy figure. In this pre-post test, as well as in the Pacman game, we use the back drivability. Indeed, because of the robot design, we have to compensate for the friction between the magnet and the ball wheel.

### 2.5. Experimental Timeline

The experiment was performed in two sessions in our laboratory at the EPFL Lausanne Campus or through two sessions at the EPFL Campus Biotech in Geneva. During the experiment, participants were sitting comfortably in front of a table with their dominant hand positioned on the table to play the game. The game rules were made known to participants: “There are six apples on the map; in order to collect the apples, you should move your robot along the paths and come to the apple. Your robot will come in front of you before the game starts. In order to finish the game, you should collect all the apples with your robot in any order you want. There will be one or two ghost robots chasing you. You should collect the apples without being caught by the ghost and without crashing into the walls. If you are caught by the ghost, all of your collected apples are eaten by the ghost and you have to recollect them to finish the game. Sometimes you can lose one apple if you crash into a wall. We will tell you when you have this rule.”

**Figure 3:**
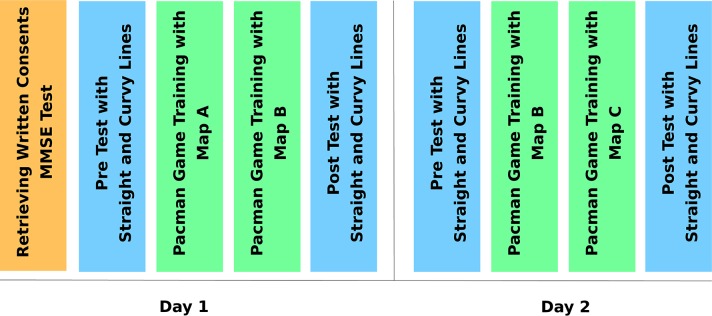
Experimental timeline.

Each participant played 53 games across three different maps (see the maps in Figure 2) within 2 consecutive days of experiments with changing game configurations and increasing difficulty per map. Each day was split into two sub-sessions. In 2 days, each participant had four sub-sessions in total. The overall experimental timeline can be seen in Figure 3. The set of repeating game configurations throughout the four sub-sessions were conceptualized as game chunks. In Table 2, the game configurations having blue color are the repeated game configurations for each map type. Within this study, these gameplay blocks of 11 games have been referred to as game chunks.

**Table 2:** Game configurations per day per map.

**Day 1 Map A**	**Day 1 Map B**	**Day 2 Map B**	**Day 2 Map C**
1, 20, –	1, 60, –	1, 20, –	1, 60, –
1, 40, –	1, 60, –	1, 40, –	1, 60, –
1, 60, –	1, 60, H	1, 60, –	1, 60, H
1, 60, –	1, 60, H	1, 60, –	1, 60, H
1, 60, H	1, 60, P	1, 60, H	1, 60, P
1, 60, H	1, 100, P	1, 60, H	1, 100, P
1, 60, P	1, 60, T	1, 60, P	1, 60, T
1, 100, P	1, 100, T	1, 100, P	1, 100, T
1, 60, T	2, 60, –	1, 60, T	2, 60, –
1, 100, T	2, 100, –	1, 100, T	2, 100, –
2, 60, –	2, 100, T	2, 60, –	2, 100, T
2, 100, –	2, 100, T, P	2, 100, –	2, 100, T, P
2, 100, T	2, 100, T, P, H	2, 100, T	2, 100, T, P, H

*Each configuration consists of the number of ghosts (1 or 2), speed of the ghost(s) in mm/s (20, 40, 60, or 100), and the applied rules (H, Haptic rule; P, Penalty rule; T, Turn rule; –, No rule). Blue colored texts represent the repeated game configurations that are defined as chunks*.

Each participant started the experiment each day with pre-tests consisting of the two line-following activities. We started from the easiest game configuration and progressively increased the difficulty by introducing a new game element one at a time, as can be seen in Table 2. We started with 20 mm/s ghost speed for the very first game of each day for each user, which we then set to 40, 60, and 60 mm/s for the next three games. Next, the assistive haptic feedback was enabled, and two more games with 60 mm/s ghost speed were played. Following that, the cross-border penalty rule was enabled and two more games were played with 60 and 100 mm/s ghost speed. Next, the turn rule was introduced, and two more games were played with 60 and 100 mm/s ghost speed. Finally, a second ghost was introduced and three more games were played with 60 and 100 mm/s ghost speed, and then 100 mm/s ghost speed was played with the turn rule, which marked the end of the games (total of 13) played with the first map.

For the second map, all above configurations except the first two (with 20 and 40 mm/s ghost speed and no rules) were repeated. After this repetition, two extra games were played with the hardest configuration, which includes the cross-border penalty rule, turn rule, 100 mm/s ghost speed, and two ghosts (again, a total of 13 games), which marks the end of the first day. In the second day, the aforementioned games were repeated. After the training session with the games, each day, the line-following activities were repeated as a post-test for the day. For the first day only, a 27th game was played with an easy configuration for the purposes of measuring the presence of any overnight learning effect.

The map order had an ABBC design, where each participant played with two of the three maps on the first day (see maps in Figure 2). By starting from the last map of the first day, the participant continued with a third map on the second day (the order of the maps is changed for each person). The overall daily game configurations and gameplay order can be seen in Table 2.

### 2.6. Data Analysis

During the gameplay, the position (*x, y*) and the orientation (θ) of each robot were recorded with around 93 Hz and sub-mm accuracy, including the robot controlled by the user. All events and interactions within the game (e.g., fruit collection, kidnapping of the robot from the map, and wall crashes) were also recorded with their time stamps. Pre-post test data and gameplay data were preprocessed using Python 3. Continuous robot pose data were filtered by the start and end time of each gameplay. Some of the participants had initial pauses in some games even though the game had already started, and in these cases we filtered game start times by counting the first movement of the user as the start time instead of the time when the ghost(s) had started to chase the Pacman.

The performance-related metrics of a participant's gameplay motion were calculated using the end-effector (hand) position data of the participant's playing hand, which was the hand holding the robot.

**Figure 4:**
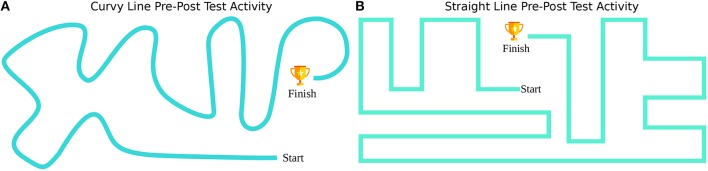
Pre-Post tests served as a measure to score transfer of game learning effects to simple robot manipulation. **(A)** is curvy trajectory line with smooth turns and **(B)** is straight trajectory line with sharp turns. The participants were instructed to complete the path from “start” to “finish” by following the respective line.

As our primary outcome, we defined a performance index. The index encompassed a metric of speed as well as accuracy to account for their tradeoff, which is often observed in behavioral (Heitz, 2014) and motor skill learning tasks (Reis et al., 2009; Ana et al., 2014). The performance index was defined as *Performance* = 1/(*Deviance*_Mean*Time_*Total*). The *Time*_*Total* metric was our surrogate for speed and was defined by the total time to complete a game, namely, a pre-post test. Our accuracy metric (*Deviance*_*Mean*) was defined by the mean Euclidean distance between the performed and optimal movement trajectory. For the Pacman game, this optimal trajectory was estimated as the middle line of each path to account for the rectangular structure of the game maze. For the pre-post tests, optimal trajectories are the given straight and curvy lines as in Figure 4.

To investigate the motor learning through time, we focused on overall learning over 2 days, online learning within each day, overnight learning between 2 days, and the transfer motor learning to a simpler activity. Overall performance for research question RQ1 was analyzed by considering the difference between all game performances of all users on day 1 and all game performances of all users on day 2.

For research question RQ2, online learning for each day was measured by testing the difference between all repeated game performances of all users in the first and the second game chunk of the corresponding day. In order to measure combined online learning of both days, we defined a term chunk order. Since there were two chunks in both days, the chunk order of the first chunks of each day was defined as 0, and the chunk order of the second chunks of each day was defined as 1.

Offline learning for research question RQ3 is supposed to be calculated as the difference between the very last performance on day 1 and the very first performance on day 2. However, this comparison has a limitation in our study design. We selected the 60 mm/s condition as the last game of the first day to test the overnight effect and the third game of the second day since they have the same configuration.

Transfer learning for research question RQ4 was measured by the performance metric calculated by pre-post tests. For research question RQ4A, overall learning in the transfer activity was measured by testing the difference in performance changes between the very first pre-test and the very last post-test. For research question RQ4B, overnight learning in the transfer activity was measured by testing the difference in performance changes between the post-test of day 1 and the pre-test of day 2. In the analysis of the curvy pre-post test, one elderly participant's data was excluded because of the missing data of a curvy pre-test of day 1.

In order to answer research question RQ5A, we investigated the effect of game elements, including the map type, speed of the ghost, number of ghosts, turn rule, haptic rule, and penalty rule, on game performance. We also investigated the effect of combinations of the game elements for research question RQ5B by checking the performance differences between game configurations across time. Since each chunk was composed of the same set of games with the same configurations, we used chunks as a measurement of time to explore the performance difference across time.

### 2.7. Optimal Game Trajectory

In tasks in which the primary outcome is based on time, e.g., in race driving, the optimal strategy for going around corners is to follow a curved trajectory. Specifically, a race driver would choose an optimal trajectory by optimizing between the shortest track and the one which would allow the highest speed, normally the one with the lowest curvature (Braghin et al., 2008). Unlike the race scenario, our Pacman game relied on speed and accuracy equally. Potentially fast curved trajectories at the corners increase the risk of hitting a wall, especially in a design in which the agent – the Cellulo robot – is already occupying a large majority of the path. For a realistic depiction of the special constraints see Figure 1.

In the design of our maps, the path is just wide enough for the robot, which visually does not provide room for the participant to cut the corner. For this reason, we have estimated the optimal trajectory as the middle line of each path. However, due to the nature of human arm motion, our method to estimate the optimal trajectory-the middle line of each path–might slightly deviate from the theoretical optimal trajectory, which might be to some extent curved in the corners. In order to see whether the mean deviance around the corners is different than the overall deviance throughout the game, we did an analysis for justifying the current analytical approach. First, we took the data around the corners in the map (turnovers in the map maze) and calculated mean deviance of only corner data. Corner data corresponded to 33% of the overall data. We then compared the corner deviance with the overall deviance by checking the group interaction with the corners. We found no significant difference between corner deviance and overall deviance and no interaction between corner and the age. These results indicated that there was not an optimal path that participants were taking around the corners.

### 2.8. Statistical Analysis

To account for the repeated measures within the participant for pre-post test measures and game performance, we used a multilevel approach for analyzing the data. Specifically, we used a hierarchical linear model (HLM) with a test time or game at the first level and participant at the second level. Using HLMs, each main and interaction effect was reported as a *t*-value. In other words, a single model can produce multiple *t*-value comparisons that are corrected for within the model. For all comparisons, the *p*-value was set at 0.05, and we measured the effect size with Pearson's correlation coefficient (r), where 0.1 is considered a small effect size, 0.3 a medium effect size, and 0.5 a large effect size.

To conduct follow-up analyses to the HLMs for specific comparisons, we conducted *t*-tests for binary comparisons and ANOVAs for multiple comparisons. For all *post-hoc* analyses, we applied a Bonferroni correction. In contrast to the *t*-test and HLM, for the effect size for the ANOVA, we computed η2, where 0.01 is considered a small effect size, 0.06 a medium effect size, and 0.14 a large effect size.

All of the HLMs were implemented in the R version 3.5.3 using package nlme version 3.1-137 (Pinheiro et al., 2020). To implement the HLMs used for the analysis, we used the linear mixed-effects models (lme) function within the nlme package. The lme function makes the assumption that the residual error and the random effects in the model are normally distributed. Within the lme function, we fitted the model using maximum likelihood. In the analysis, for all *t*-tests that were conducted, we used the *t*-test function in the R package stats version 3.5.3. Finally, to conduct an ANOVA that accounts for the repeated measures of the data, we used a combination of the R packages lme4 version 1.1-21 (Bates et al., 2015), stats version 3.5.3, and psycho version 0.4.0 (Makowski, 2018). In the lme4 package, we used the function lmer to build the model. Like the lme function, the assumptions are that the residual error and the random effects in the model are normally distributed. We conducted an ANOVA on the model using the anova function from the stats package. Finally, we used the psycho package to conduct a *post-hoc* analysis on the contrasts using the get_contrasts function. For all figures, error bars represent the standard error.

## 3. Results

### 3.1. Overall Learning (Across Day Game Performance)

**Figure 5:**
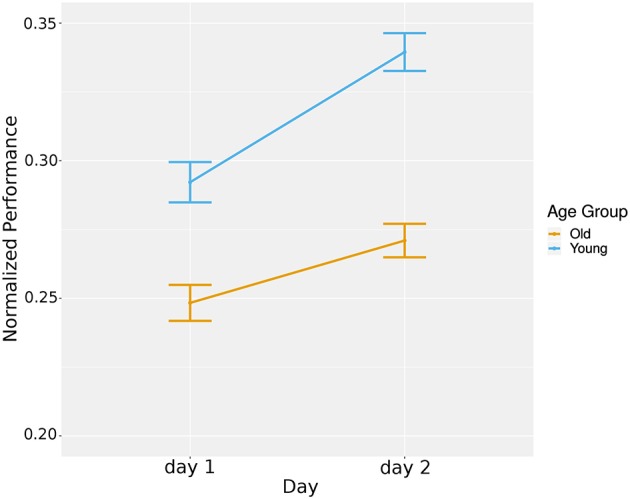
Overall learning: averaged normalized game performance plotted per day per group.

To investigate the differences between day 1 and 2 game performance and how the age of the participant may impact the performance, we ran a hierarchical model to account for the repeated measures design. At the first level, we took into account the time when a participant was interacting with the Pacman game. At level two, we accounted for the individual participants. To control differences in the game configurations, we included the game features as covariates in the model. This included the map type, speed of the ghost(s), the number of ghosts, haptic rule, penalty rule, and turn rule. We found a significant main effect for time, *t*_(1705)_ = 2.93, *p* < 0.01, *r* = 0.07, with participants overall having a higher performance on the second day (*M* = 0.27, *SD* = 0.15 for day 1, *M* = 0.30, *SD* = 0.14 for day 2). We also found a main effect for age group, *t*_(31)_ = 2.18, *p* < 0.05, *r* = 0.36, with young group having a higher performance in both days compared to the elderly (*M* = 0.32, *SD* = 0.15 for young, *M* = 0.26, *SD* = 0.14 for elderly). Additionally, there was a significant interaction between time and age group, *t*_(1705)_ = 2.56, *p* < 0.05, *r* = 0.06, with the performance change being stronger in the young age group (see Figure 5). To investigate if the learning of both groups was significant, we repeated the tests separately for each group and found significant effect of time in the old group, *t*_(874)_ = 2.97, *p* < 0.01, *r* = 0.1 and in young group, *t*_(822)_ = 6.45, *p* < 0.001, *r* = 0.23.

In summary, both age groups improved in game performance over time, with the young group demonstrating higher performance levels and a larger slope of improvement.

### 3.2. Online Learning

To analyze if there was online learning within the participants, we conducted a hierarchical linear model to take into account the repeated games for each participant. At the first level, we included day, chunk order, and age group to test their effects on the normalized performance. To allow for an equal comparison both within a day and between days, we analyzed only the game chunks that were consistent across days.

We did find a trend for the main effect of order or age group, *t*_(1413)_ = 1.83, *p* = 0.07 and *t*_(31)_ = 1.71, *p* = 0.09, respectively. We did find a significant main effect of day, *t*_(1413)_ = 3.02, *p* < 0.01, *r* = 0.08, with day 2 having better performance than day 1 (*M* = 0.28, *SD* = 0.15 for day 1, *M* = 0.31, *SD* = 0.14 for day 2), which was consistent with our overall learning results that assessed all games played in a day rather than only games in repeated chunks.

In terms of the interaction effects, we found a significant interaction between chunk order and day, *t*_(1413)_ = −2.39, *p* < 0.05, *r* = 0.06, with the online learning occurring on day 1 being significantly more positive than the learning that occurred on day 2. There was not a significant interaction between age group and chunk order or day, *t*_(1413)_ = 0.39, *p* = 0.69, and *t*_(1413)_ = 1.42, *p* = 0.15, respectively. For the third order effect, we did not find a significant difference, *t*_(1413)_ = 0.05, *p* = 0.96.

**Figure 6:**
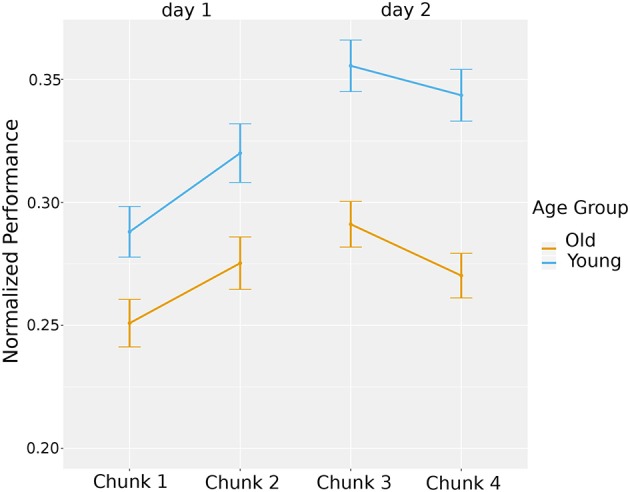
Online learning: normalized game performance plotted per group per chunk (repeating games in each subsession).

To investigate if the learning within each day was significant, we ran two paired *t*-tests comparing chunk performances within each of the days separately. We found significant learning gains in day 1, *t*_(362)_ = 2.98, *p* < 0.01, *r* = 0.15, with a mean difference of test times being 0.028, but we did not find a significant decrease in learning in day 2, *t*_(362)_ = 1.84, *p* = 0.07, with the mean difference of test times being −0.017 (see Figure 6). Overall, across both age groups, stronger online effects were present on day 1.

### 3.3. Offline Learning

**Figure 7:**
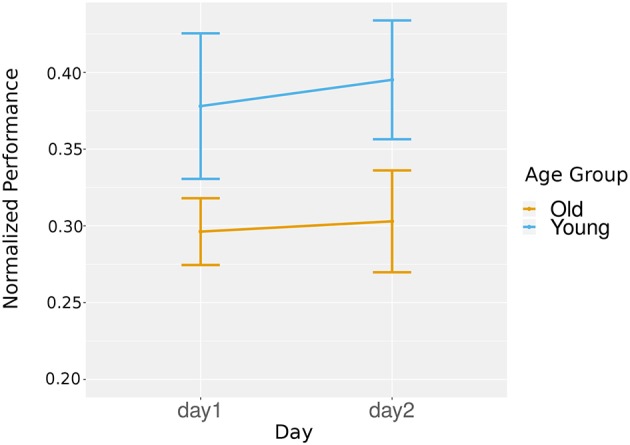
Offline learning: averaged normalized game performance in offline probe games per group (1 Ghost 60 mm/s, last game of day 1 and third game of day 2).

To address whether or not there was offline learning and whether or not age group had an impact on offline learning, we conducted a hierarchical model to account for the repeated measures design with two game data being recorded for a single participant. Specifically, we compared the performance on the last game of day 1 and the third game of day 2, as they had the same configurations. We did not find a significant main effect for day or age group, *t*_(31)_ = 0.16, *p* = 0.87 and *t*_(31)_ = 1.60, *p* = 0.12, respectively. Additionally, we did not find a significant interaction between day and age group, *t*_(31)_ = 0.18, *p* = 0.86 (see Figure 7). This indicated that no offline enhancement was present in either age groups.

### 3.4. Impact of the Game Elements

#### 3.4.1. Effect of Each Game Element on Game Performance

**Figure 8:**
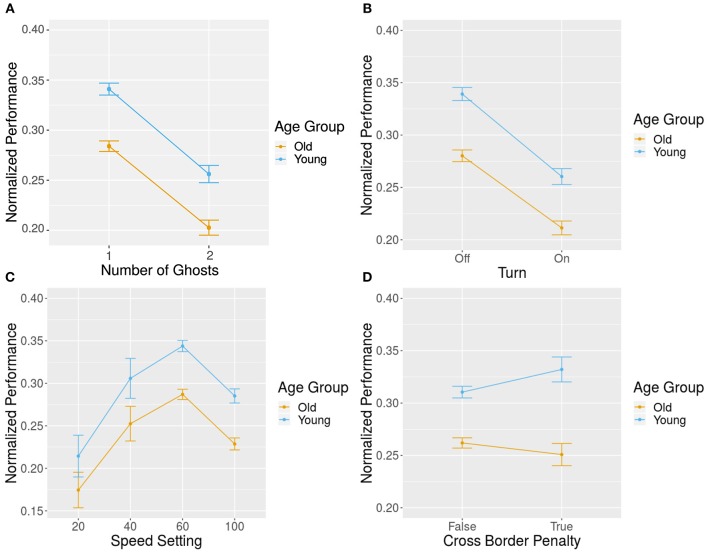
Impact of individual game elements on normalized performance: effect of **(A)** number of ghosts, **(B)** speed in mm/s, **(C)** turn rule, **(D)** cross-border penalty rule.

Several elements can be adapted within a game setting that might change the user's performance. We investigated the effect of game elements, including map type, speed of the ghost, the number of ghosts, turn rule, haptic rule, and penalty rule, on the performance and their interactions with the age groups. To investigate these effects, we ran a hierarchical model to account for the repeated measures design. At the first level, we considered the game configurations. At level two, we accounted for the individual participants as each participant played multiple games. We found a significant main effect of the number of ghosts (*M* = 0.31, *SD* = 0.14 for one ghost, *M* = 0.23, *SD* = 0.13 for two ghosts), ghost speed (*M* = 0.19, *SD* = 0.13 for 20 mm/s, *M* = 0.28, *SD* = 0.13 for 40 mm/s, *M* = 0.31, *SD* = 0.14 for 60 mm/s, *M* = 0.26, *SD* = 0.14 for 100 mm/s), turn rule (*M* = 0.31, *SD* = 0.15 for turn rule is off, *M* = 0.24, *SD* = 0.12 for turn rule is on), and orange map (*M* = 0.26, *SD* = 0.12 for orange map, *M* = 0.29, *SD* = 0.14 for green map, *M* = 0.31, *SD* = 0.17 for yellow map) (see Table 3, Figures 8A–C).

**Table 3:** Impact of individual game elements.

**Game element**	**Main effect**	**Age group interaction**
2 Ghosts	*t*_(1698)_ = −7.78, *p* < 0.0001, *r* = 0.19	*t*_(1698)_ = 0.04, *p* = 0.97
Speed 40	*t*_(1698)_ = 2.69, *p* < 0.01, *r* = 0.07	*t*_(1698)_ = 0.32, *p* = 0.75
Speed 60	*t*_(1698)_ = 6.07, *p* < 0.0001, *r* = 0.15	*t*_(1698)_ = 0.49, *p* = 0.63
Speed 100	*t*_(1698)_ = 6.07, *p* < 0.0001, *r* = 0.15	*t*_(1698)_ = 0.29, *p* = 0.77
Turn	*t*_(1698)_ = −6.08, *p* < 0.0001, *r* = 0.15	*t*_(1698)_ = −0.57, *p* = 0.57
Haptic wall	*t*_(1698)_ = 1.04, *p* = 0.29	*t*_(1698)_ = 0.12, *p* = 0.9
Cross border penalty	*t*_(1698)_ = −1.89, *p* = 0.06	*t*_(1698)_ = 2.36, *p* < 0.05, *r* = 0.06
Orange map	*t*_(1698)_ = −4.15, *p* < 0.0001, *r* = 0.1	*t*_(1698)_ = −0.35, *p* = 0.73
Yellow map	*t*_(1698)_ = −0.58, *p* = 0.56	*t*_(1698)_ = 1.26, *p* = 0.21

*Blue font indicates p < 0.05*.

For differences in the age group, we only found an interaction in the cross-border penalty rule (see Table 3 and Figure 8D). In order to investigate the effect of cross-border penalty, we did a separate analysis for each age group. We found a trend for the main effect of cross-border penalty in the elderly group *t*_(874)_ = −1.95, *p* = 0.05 while we did not find a significant effect of cross-border penalty in the young group *t*_(822)_ = 1.42, *p* = 0.15.

In summary, most game elements had a significant main effect but showed similar performance differences in both groups. However, the cross-border penalty rule was the only game element with a differential effect on age group. When enabled, it resulted in a trend of disturbed performance in the elderly, but it did not affecting the performance of the young group significantly.

#### 3.4.2. Effect of Game Configurations on Learning

To investigate the learning that occurred within each game configuration not just the impact of the individual elements on performance, we compared the performance on different game configurations across time. Since each chunk consisted of the same set of games, we used chunks as a measurement of time. Additionally, because each game configuration had a different hardness level, we normalized the performance of game play for each configuration type by its baseline performance (i.e., the performance in chunk 1). This normalization allowed us to compare learning between configuration types without the influence of hardness.

We ran an HLM to analyze the differences between chunk and age group while accounting for the repeat of chunk within users. We found that chunk 2, 3, and 4 all had greater performance than the baseline, chunk 1, *t*_(1413)_ = 4.08, *p* < 0.05, *r* = 0.11, *t*_(1413)_ = 5.75, *p* < 0.05, *r* = 0.15, and *t*_(1413)_ = 3.83, *p* < 0.05, *r* = 0.10, respectively (*M* = 0.27, *SD* = 0.14 for baseline chunk 1, *M* = 0.30, *SD* = 0.15 for chunk 2, *M* = 0.32, *SD* = 0.14 for chunk 3, and *M* = 0.31, *SD* = 0.14 for chunk 4). There was not a main effect of age group, *t*_(31)_ = 0.00, *p* = 0.99. Additionally, there was no significant interaction between age groups and chunks, *t*_(1413)_ = −0.98, *p* = 0.33 (chunk 2), *t*_(1413)_ = −0.86, *p* = 0.39 (chunk 3), and *t*_(1413)_ = −0.21, *p* = 0.84 (chunk 4).

To investigate how the learning may have changed between any two chunks, we ran an ANOVA to account for the specific differences. To better delineate the learning that may occur within each age group, we ran the analysis for the young and old users separately. For each age group, we used a repeated measures ANOVA to account for the repeated chunks for each user. For any significant results, a *post-hoc* analysis was used to assess the contrasts.

For the young users, we found a significant main effect of configuration (*F*_(8, 653)_ = 2.01, *p* < 0.05, η2 = 0.22) and a significant main effect of chunk [*F*_(3, 653)_ = 13.18, *p* < 0.05, η2 = 0.58]. We did not find a significant interaction between configuration and chunk [*F*_(24, 653)_ = 0.60, *p* = 0.94]. In a *post-hoc* comparison, the only significant difference between configurations was between the configuration with “1 Ghost 100 Speed Turn On” and the configuration with “2 Ghosts 100 Speed Turn On,” which is the hardest configuration in the chunk [*t*_(653)_ = 3.70, *p* < 0.01, *r* = 0.14].

In the *post-hoc* analysis of chunks, we found a significant difference between chunk 1 and chunk 2, 3, and 4 (as was the case in the overall analysis), *t*_(653)_ = −3.38, *p* < 0.01, *r* = 0.13, *t*_(653)_ = −5.99, *p* < 0.001, *r* = 0.23, and *t*_(653)_ = −4.64, *p* < 0.001, *r* = 0.18, respectively. Additionally, there was a significant difference between chunk 2 and chunk 3 [*t*_(653)_ = −2.61, *p* < 0.05, *r* = 0.10].

Similar to the young users, for the old users, we found a significant main effect of configuration [*F*_(10, 688)_ = 2.83, *p* < 0.05, η2 = 0.41] and a significant main effect of chunk [*F*_(3, 688)_ = 8.14, *p* < 0.05, η2 = 0.35]. We did not find a significant interaction between configuration and chunk [*F*_(30, 688)_ = 0.58, *p* = 0.97]. In a *post-hoc* comparison, we found two significant differences between game configurations, both including configuration with “2 Ghosts 100 Speed Turn On.” The significant game differences included configuration with “1 Ghost 100 Speed Penalty On” [*t*_(688)_ = 3.44, *p* < 0.05, *r* = 0.13] and configuration with “1 Ghost 100 Speed Turn On” [*t*_(688)_ = 3.67, *p* < 0.05, *r* = 0.14].

In the *post-hoc* analysis of chunks, we found a significant difference between chunk 1 and chunk 2, 3, and 4 (as was the case in the overall analysis), *t*_(688)_ = −3.39, *p* < 0.01, *r* = 0.13, *t*_(688)_ = −4.77, *p* < 0.001, *r* = 0.18, and *t*_(688)_ = −3.18, *p* < 0.01, *r* = 0.12, respectively.

**Figure 9:**
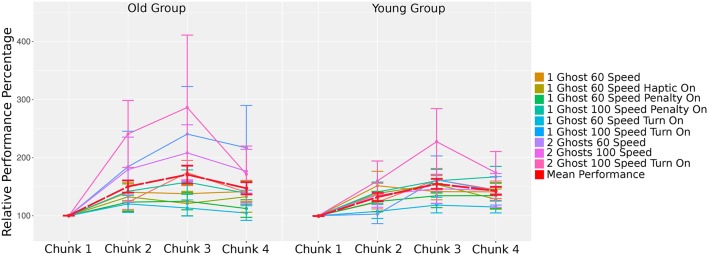
Relative performances: average game performance normalized to Chunk 1 of each group.

In summary, the results confirm that both age groups demonstrated learning over time in all applied game configurations. Performance significantly increased until the first session of day 2 for both groups, and the performance level stayed similar until the last session on day (see Figure 9). Similar to the previous results, age difference was very slight, which is related to the cross-border penalty rule effect on elderly.

### 3.5. Transfer Learning

#### 3.5.1. Overall Learning in Transfer Activity

To address research question RQ4A, if there was transfer learning for the participants in both line following activities, we ran two hierarchical linear models to take into account the repeated tests within the participant. For each line we did a separate analysis since the performance value for each line following activity was different due to the design differences. We did not find a significant main effect of age group for curvy and straight lines, *t*_(31)_ = 1.25, *p* = 0.23 and *t*_(31)_ = 1.18, *p* = 0.25, respectively.

We did find a significant main effect of day in the curvy test, which was overall learning from pre-test of day 1 to post-test of day 2, *t*_(30)_ = 2.24, *p* < 0.05, *r* = 0.38, with the last post-test having higher performance than the very first pre-test (*M* = 0.19, *SD* = 0.11 for the curvy pre-test of day 1, *M* = 0.27, *SD* = 0.10 for the curvy post-test of day 2). Similarly, a significant main effect of day was found in the straight test, which was overall learning from pre-test of day 1 to post-test of day 2, *t*_(31)_ = 3.92, *p* < 0.001, *r* = 0.58, with the last post-test having higher performance than the very first pre-test (*M* = 0.08, *SD* = 0.05 for straight pre-test of day 1, *M* = 0.16, *SD* = 0.07 for straight post-test of day 2).

#### 3.5.2. Overnight Learning in Transfer Activity

**Figure 10:**
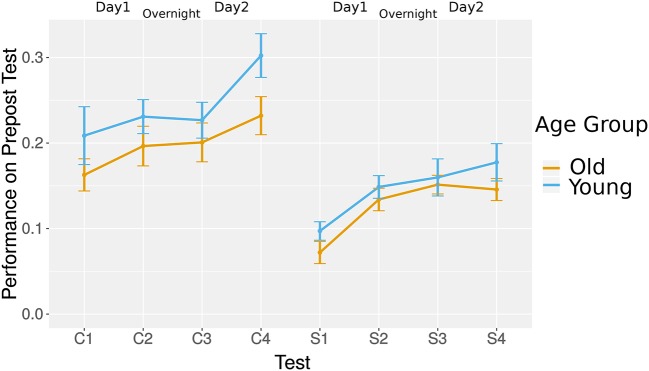
Transfer: average performance in pre and post-tests. C, curvy trajectory; S, straight trajectory and angled trajectory.

To address research question RQ4B, we investigated overnight learning in transfer activity by comparing the post-test of day 1 and pre-test of day 2. As with the overall transfer learning, we ran two HLMs, one for each line type. We did not find a significant offline learning in curvy and straight tests, *t*_(31)_ = 0.2, *p* = 0.85 and *t*_(31)_ = 1.42, *p* = 0.17, respectively (see Figure 10).

These results indicated that game-based learning transferred to simple robot manipulation measured with the applied straight line test (which is similar to the Pacman maze) but also with the curvy line (which is closer to the daily activity motions). The performance levels and learning were similar in both age groups.

## 4. Discussion

The present project was designed to determine whether healthy elderly subjects are able to utilize and acquire novel motor skills through the use of the Cellulo Pacman game. Furthermore, we investigated the impact age exerts on different features of learning. One main finding was that elderly participants were able to improve with the usage of (and thus learn) the Cellulo Pacman game over time. This was evident by an overall significantly improved game performance. Importantly, game-based learning was transferred to simple, non-trained robot manipulations, which emphasized the Cellulo interface as a potential tool to conduct motor training regimes to promote healthy aging or support motor rehabilitation in pathological conditions. Age-related differences were apparent during the learning process, such as a reduced overall game performance and the effect of cross-border penalty rules.

### 4.1. Overall Learning

Elderly participants presented lower performance levels and smaller overall performance gains when training with the Cellulo Pacman task. This resembles a frequently described pattern of the elderly population throughout a variety of motor tasks, e.g., reduced performance gains in fine motor skill learning (Voelcker-Rehage, 2008; Zimerman et al., 2013). The extent of these age-related differences has been shown to be dependent on several factors, such as task structure, complexity, difficulty, and the familiarity level (Voelcker-Rehage, 2008). A possible underlying mechanism could be a reduction in neuroplastic capacities in the elderly population, based, for instance, on age-related deficits in long-term potentiation (Watson et al., 2002; Di Lazzaro et al., 2008).

### 4.2. Online Learning

Subsequently, we assessed different temporal components of the learning process. Both groups showed similar characteristics in online learning. Interestingly, online learning on day 1 (chunk 2 vs. chunk 1) was significantly higher than online learning on day 2 (chunk 4 vs. chunk 3), indicating that there was manifest online learning within the first day for both groups. On the contrary, there was no manifest learning, but also no forgetting, on the second day. Several factors could explain this pattern, such as fatigue, novelty effect, or ceiling. A supporting argument for the fatigue hypothesis could be that, during the first chunk of day 2, the participants started at a higher performance level compared to the first chunk of day 1. They may thus have recruited more endurance resources early during the daily session and may have reached their physical exhaustion phase faster on day 2 (Finneran and O'Sullivan, 2010). It is shown that motor learning procedures lead to better results when the practice is done over several days or even weeks (Shea et al., 2000). Therefore, rather than having a large amount of exercise, distributing practice trials over several days may result in better improvement (Baddeley and Longman, 1978; Savion-Lemieux and Penhune, 2005; Blischke and Erlacher, 2007). In our trial sessions, resting times between trials and between chunks were short and daily sessions were intense. In order to provide a better training regime, breaks between games could be increased, and sessions could consist of fewer games.

Secondly, the loss of gain in online performance at the end of day 2 could be attributed to a novelty effect, which postulates that novel environments and interactions promote plasticity and learning (Schomaker, 2019). This may have been saturated by the end of day 2. Novelty effects are a reported phenomenon for human robot interactions (e.g., Kanda et al., 2004).

Lastly, this discrepancy in the amount of online learning between days could be explained by ceiling effects. In general, one would expect faster ceiling in motor tasks with low difficulty compared with tasks with high difficulty (Guadagnoli and Lee, 2004). Our data partially resembles this expected pattern, as the easier game configurations tend to show an early decrease in slope, when compared to the harder game configurations, such as games having 2 ghosts (represented by the lines having colors closer to pink), see also Figure 9.

### 4.3. Offline Learning

Sleep has been shown to benefit many processes of learning and memory, and may also have an important role in the homeostatic regulation of neural mechanisms (Walker et al., 2002; Gudberg and Johansen-Berg, 2015). After the initial online learning, sleep can enhance the performance level of procedural motor skills (Walker et al., 2002). Mechanistically, the amount of stage 2 NREM sleep (Walker et al., 2002) and the local increase of slow wave activity in parietal regions has been associated with the amount of offline learning (Huber et al., 2004). These and complementary findings led to the postulation of the sleep-based enhancement hypothesis. This sleep-based enhancement (offline learning) has been shown to be impaired in the elderly population (e.g., Spencer et al., 2007).

We did not observe offline learning for either the young or elderly group in the present game-based task. However, there was also no loss of skill (forgetting) after the overnight interval. Our findings are in line with current findings that suggest that the classically described sleep-dependent offline gains are dependent on the task demands, task condition, and phase in lifespan (Adi-Japha and Karni, 2016). Moreover, in addition to sleep-dependent effects, different confounding variables, namely the time of day of testing, confounds introduced by data averaging, or performance-to-break ratio, have been discussed (Pan and Rickard, 2015). We speculated that the task-nature and the rather low complexity (Blischke and Malangré, 2017) of the offline probe prevented the evolution of positive offline learning effects in the young cohort. The absence of offline losses, previously described in elderly (Spencer et al., 2007), might be explained by the study design, since we employed two easy “warm-up” game configurations on day 2 first before scheduling our offline learning probe (game configuration 1 ghost 60 mm/s). Since we focused on performances related to motor learning measured by accuracy and total time, the present result might not reflect other aspects of the game that might be improved overnight.

### 4.4. Impact of the Game Elements

#### 4.4.1. Effect of Each Game Element on Game Performance

The effects of game elements, such as the number of ghosts, ghost speed, and turn rule, on the performance were similar in both groups. Only slight age-related differences were apparent for the application of the cross-border penalty rule, which had a larger impact in the elderly population.

The effect of the cross-border penalty rule on elderly participants' different performance metrics was analyzed to understand the reason behind this group difference. We observed an increase in mean accuracy, a decrease in mean velocity, and therefore, an increase in total time to complete the game. The cross-border penalty rule increases the importance of accuracy during the game. Therefore, it might result in a decrease in the speed. One of the reasons for this age-related difference might be that older adults are reluctant to make mistakes; they value accurate responses over responding quickly (Rabbitt, 1979; Smith and Brewer, 1995; Starns and Ratcliff, 2010; Forstmann et al., 2011). As a consequence, in order to circumvent mistakes and to reach a better performance, older adults adjusted a balance between the opposing demands of accuracy and speed, resulting in a relatively slow performance (Rabbitt, 1979; Smith and Brewer, 1995; Ratcliff et al., 2007; Starns and Ratcliff, 2010; Forstmann et al., 2011).

Adding an extra ghost, turn rule, and increasing the speed to 100 mm/s in the game decreases the performance of the users. The possible explanation to that decrease could be the increase in the hardness or the challenge of the game. Speed of enemies, frequency of enemies, power of player (e.g., skills, speed, and tools), and duration of game-play experience are the most common game elements with which to define or adjust the difficulty of a game (Sicart, 2008; Adams and Dormans, 2012). Similarly, in our proposed Pacman game, these elements corresponded to the speed of the ghost, number of ghosts, speed of the user (which slows down with the turn rule to collect the apple by rotating the robot), and total time to collect six apples. When the ghost speed increases to 100 mm/s, a second ghost is added to the game, or a turn rule is added to the game; the probability of being caught by the ghost thus increases, and the user should run away from the ghost faster, which might result in them not collecting apples in a time-efficient order. The user also should be more attentive toward the ghosts in these cases since the probability of being caught increases. This might also decrease the attention toward walls and the overall accuracy of the motion may decrease. On the other hand, increasing the speed from 20 to 40 mm/s and 60 mm/s pushes the user to perform better. The reason behind this result might be that increasing the speed from 20 to 60 mm/s increases the user's speed without making game challenging enough to perform worse.

The relationship between ghost speed and performance might also be explained by the Challenge Point Framework proposed by Guadagnoli and Lee (2004). Increases in functional task difficulty (e.g., increasing ghost speed) is expected to result in decreased performance; however, depending on the skill level of the performer, the optimal challenge point changes. The decrease in the performances of the participants after 60 mm/s ghost speed might show that the optimal challenge point in Pacman game for healthy users starts with ghosts having 60 mm/s speed.

Map design also affects the performance of the user. Even though the yellow map was smaller in range of motion, the overall performances on the yellow map and green map were similar. This result might imply that the yellow map provided as much challenge as the green map. This challenge can be explained with the limited space for running away from the ghosts. On the other hand, even though the sizes of the green and orange maps are the same, performance of the users on the orange map was less than the green one. This can be due to the internal maze and connection design. A more detailed challenge analysis will be conducted in future studies.

#### 4.4.2. Effect of Game Configurations on Learning

Following the effect of each game element, we also assessed the learning behavior in more detail by separating learning curves for each individual repeating game configuration to investigate the effect of configurations on learning between game chunks.

The effect of different configurations on learning between chunks were found to be similar in both groups; we did not see any group level differences in either group. It is of note, that we accounted for the easiest and hardest games in the overall learning, but not in the online learning, since the online analysis was based on chunks. This created the existing group effect in overall learning. There was an increase in performance from the very first chunk to the last chunk. Similarly to the previously discussed online learning results, in the relative performance change per configuration, we also found a significant performance increase within day 1, while there was no significant learning or forgetting within day 2, as can be seen in Figure 8.

When we compared each configuration to the other configurations, in both groups, the hardest game with turn, two ghosts, and maximum speed had the higher increase in learning, while the game with turn, one ghost, and maximum speed had the lowest improvement compared to their own baseline level. In both groups, there was a significant difference between these two configurations. The reason behind this result might be that, when the turn rule is applied while the ghost is fast, compensating for the total time or speed of the game could be harder because of the nature of the turn rule. However, the same condition with two ghosts can be improved by providing strategies against two ghosts over time.

The only difference between groups was that the elderly group had a significant difference between the hardest condition and the penalty rule in the fast ghost condition. These results are coherent with the results of the effect of game elements on the performances where we observed no group differences for several game elements except the wall crash penalty rule. The reasons for this age-related difference might be that older adults are more reluctant to make mistakes and that the learning process of a game where the user has to be accurate under the high speed forced by the ghost is prolonged.

### 4.5. Transfer Learning

To determine whether the acquired skills during the game have any impact on daily life, we tested the transfer of these improvements to non-trained simple tasks similar to daily life activities. Both groups improved in the robot manipulation task on both straight and curved trajectories and thus showed a clear transfer of the acquired motor skill. Contrary to the game performance difference between groups, the performance level and the improvement rate in transfer learning were similar for both groups. This might be due to the simplicity of the transfer activity, which does not require a high cognitive effort rather than simple physical manipulation.

This successful transfer to simple robot manipulation is an important first hint that is indicative of the translational potential of the Cellulo system into an evidence-based motor training tool to promote healthy aging. Simple Cellulo robot manipulation resembles activities of daily living, such as using a computer mouse, wiping a table, or the general transfer of objects in the horizontal plane. Ultimately, translation of improvements in a robot-assisted motor therapy to measure upper limb capacity, e.g., the Action Research Arm Test (ARAT) or Wolf Motor Function Test (WMFT), and measures of basic activities of daily living will be the crucial benchmark for successful translation toward real life situations. In this regard, the current effectiveness of available robot-assisted motor training systems addressing the upper limb remains still limited (Veerbeek et al., 2017).

### 4.6. Future Directions

In the current study, the potential effects of robotic motor training and gamification were not assessed separately in individual control conditions, preventing us from disentangling specific contributions of the individual strategies on the training effects. We aim to assess this open question in future studies.

In this study, the performance metric was calculated by movement deviation and movement time. However, the amount of motion performed by the user and its correlation with different muscle groups' activities was also important to an adaptive exercise system. We aim to address this point in our future studies focusing on motor training aspects by accounting the motion trajectories and range of motion.

Apart from the physical aspects, like motor learning, the game also included several cognitive aspects, such as remembering which apples had been collected, remembering the last apple eaten, being aware of the positions of the chasing ghosts, and creating a strategy to trick them or run away from them. These cognitive aspects will be addressed by extracting the information related to strategy of the user through gameplay data.

In order to provide an adaptive personalized exercise of activities of home usage, the system should provide in- and between game adaptations. Through our proposed game configurations, providing adaptivity between games by measuring the user's previous performance was possible. In future studies, the aim will be to design and implement changing ghost behaviors as an adaptive in the game mechanism. In addition, more studies are needed to assess the long-term impact of Cellulo robotic training on improvement in motor functions, and this is also true for the effect of gamification compared to a task-based training with the same platform. Providing social play through our platform is also another future direction that we will follow to increase the enjoyment and motivation to do gamified exercise activities at home.

Furthermore, the Cellulo system also has considerable potential to translate toward clinical applications. It provides the advantage that different behavioral domains, such as motor functions (e.g., dexterity) and cognitive functions (e.g., decision-making and planning), could be trained conjointly in an entertaining way with as system potentially suitable for home-based use. This approach may provide a benefit for several neurocognitive disorders, including Alzheimer's, Parkinson's disease, or stroke, which frequently affect both behavioral domains (Scarmeas et al., 2004; Poewe, 2008;Ramsey et al., 2017).

### 4.7. Limitations

One of the limitations of this study was that the potential of the system was shown through only one game. Other games can be designed to include reaching and catching motions without having a focus on path following, and cognitive games can be designed by focusing on attention and memory. Using the same robot within the transfer activity is also a limitation of the study. Additional assessment tool for transfer activities would have been ideal and should be used in future studies.

Another limitation of the study is related to the experimental design, which ordered games from easy to hard. An attempt was made to limit this effect via chunk design, but, still, in order to provide a smooth learning process of the game, we had to start from easier games and increase the difficulty. Apart from this, gender distribution was also not equal, and this prevented us from determining the potential effects of gender. This gender bias might also have an effect on the results. In the young and elderly groups, the distribution of education and professions were not balanced, and we did not ask if they were active or motivated “game players”. These might have an effect on the performance of the task.

Because the technology used in this study is new to motor training, it was not clear before the study what the expected effect size would be. A natural limitation that follows from the exploratory nature of our study is that we did not conduct a power analysis to avoid an inaccurate assessment based on unsupported assumptions. However, moving forward, our study can be used as an anchor point for target effect sizes for motor training with similar technologies to conduct a priori power analyses.

## 5. Conclusion

Elderly people showed learning success when training a gamified motor task with the Cellulo platform. Importantly, the game-based learning gain translated to the performance of simple robot manipulation, which resembles activities of daily life. By this, the Cellulo platform has the potential to develop into a tangible, low-cost, motor training solution with the goal of facilitating healthy aging. It complements available motor training paradigms and may also be suitable for home-based, gamified, and motivational use.

## Data Availability Statement

The datasets generated for this study are available on request to the corresponding author.

## Ethics Statement

The studies involving human participants were reviewed and approved by the Cantonal Ethics Committee Vaud, Switzerland (project number 2017-00765). The patients/participants provided their written informed consent to participate in this study. Written informed consent was obtained from the individual(s) for the publication of any potentially identifiable images or data included in this article.

## Author Contributions

AG, MW, WJ, FH, and PD: conception and design of the experiment. AG, MW, PD, and AO: collection of data. AG and MW: literature search. AG: system design and implementation. AG and JO: statistical data analysis. AG, JO, WJ, MW, FH, and PD: interpretation of the results. AG, JO, and MW: drafting of the manuscript. AG, MW, AO, FH, and PD: critical revision of the manuscript for important intellectual content.

### Conflict of Interest

The authors declare that the research was conducted in the absence of any commercial or financial relationships that could be construed as a potential conflict of interest.
